# Book Reviews

**Published:** 1848-12

**Authors:** 


					﻿BIBLIOGRAPHICAL NOTICES.
Annuaire de Therapeutique de Mature Medicate, de Pharmacie et de
Toxicologie, pour 1848 ; nontenant le Resume des Travaux Th£-
rapeutiques et Toxicologiques publies en 1847, et les Formules
des Medicaments nouveaux, suivi de nouvelles observations sur
la Glucosurie, d’une notice sur la Therapeutique des affections
syphilitiques, et d’un memoire sur 1’influence des nerfs pneumo-
gastriques dans la digestion. Par le Dr. A. Bouchakdat, Cheva-
lier de la Legion d’Honneur, Agrege de la Faculte de Medecine
de Paris, Pharmacien en Chef de 1’Hotel Dieu, &c.	24mo. pp.
312. Paris, 1848.
Handbuch der Pharmakodynamik fur Aerzte, Wundarzte und
  Studirende.  Nach der  neuesten Erfahrungen des In-und Aus-  
  landes wie  auch  nach  eigener dreissigjahriger Erfahrung am
  Krankenbette,  kritisch  bearbeitet  von MARTIN WILHELM
  PLAGGE, Med. et Chir. Dr. 8vo. s. 680.   Braunshweig, 1847.
Pl Manual of Pharmacodynamics for Physicians, Surgeons and
Students, critically prepared after the most recent experience at
home and abroad, and a thirty years experience at the bedside.
By Martin William Plagge, Doctor of Medicine and Surgery.
8vo. pp. 680. Brunswick, 1847.
The Annual of M. Bouchardat is always welcome to us. It is a
resume of what has been effected during the year on the different
departments of which it treats, and is by no means superseded by
the semi-annuals of Braithwaite and Ranking, or the periscopes
of our different Journals. The author is a skilful pharmacien,
and a zealous investigator of therapeutical agencies ; and if there
appears too much of the egomet ever and anon in his productions,
we are led to overlook it in consequence of the mass of valuable
material in which it is enveloped.
The present volume contains the wonted amount of ‘ New
Remedy’ matter; and, as usual, the researches of its author on
special subjects, to one of which, Diabetes, or, as he terms it,
Glucosurie, there appears to be no end. But we will permit him to
tell his own story in regard to the varied contents of his Annual.
“ The therapeutical discovery, which has caused the most noise
in the year, is, undoubtedly, the employment of ether and chloro-
form to suppress pain in surgical operations. I have followed with
the most scrupulous attention the progress of that great question,
and hope that my readers will find brought together, and summed
up, the most important facts that appertain to it.
“Pharmacologists have this year concentrated their efforts on the
question of purgatives. To make them evacuate in the most agree-
able manner is the problem with which they have occupied them-
selves a Penvi. I have collected and given my opinion of the pub-
lications that belong to it.
“ The Annuaire of 1838 contains a resume of the works on the-
rapeutics that throw light on questions relating to the employment of
medicines. I may particularly specify the articles that refer to the
employment of aconite, veratria, the essence of turpentine, ergot,
phosphate of ammonia, the resins of scammony and jalap, pharyngeal
cauterization with ammonia, &c.
“ Under the title of surgical therapeutics, I have given a summary
of the important labours of MM. Blandin, Boyer, Robert, and
Chassaignac, on anal tenotomy, hemorrhoidal excrescences, hospital
gangrene, and the ophthalmia of the new born.
“ I have consulted all the periodical collections. The Bulletin
de Therapeutique, especially, has been very useful to me. This
journal has lost its chief editor, M. Miquel, but in perusing carefully
the volumes for 1847, we see that they need not envy their prede-
cessors, and that M. Debout will continue worthily and honourably
a work so eminently practical.
“ My own labours occupy a large space in the volume. I may
indicate especially the article on atropia, which is common to me
and M. Stuart Cooper. It is the resume of a work which we purpose
publishing soon on that article of the materia medica, which is
destined for a high place in therapeutics.
“I trust that my readers will peruse with interest the case of in-
termittent fever, which has continued for forty-four years, and the
practical remarks on the employment of the sulphate of quinia in
intermittent maladies.
“The volume is terminated by memoirs on three different subjects.
In the last, M. Sandras and I have fixed the role of the pneumogas-
tric nerves in digestion, by showing that the section of those nerves
suspends stomachal, without throwing any obstacle in the way of
intestinal digestion. In appreciating by that experiment this great
act, we have confirmed, in the clearest manner, our preceding obser-
vations. In this way we may follow phases of digestion, which
are frequently independent of each other.
“ The two essays which have reference to glucosurie are intended
to complete my observations, as well as to reply to objections] or
critiques to which this portion of my investigations has given oc-
casion.” p. vi. &c. &c.
This is not the place to introduce the different formulae, which
M. Bouchardat has given as the result of his own suggestions, but
particularly of those of other observers. The volume itself may
properly find a place in the library of the progressive practitioner.
The author of the second work, whose title is given at the
head of this article, we regret to find, by a notice of the publisher
appended to the preface, died soon after the completion of the un-
dertaking, having left materials towards the publication of a
“ Manual of dietetical, psychical and physical therapeutical
agents ”—“ Handbiich der dicitetis chen, psychischen und physikal-
ischen Heilmittel.” These materials, we are glad to find, are not
to be lost to science, but will be transferred by the publisher to
competent hands.
M. Plagge’s work contains a large amount of useful matter ;
and is unquestionably one of the best of its class. We can give
an idea only of its varied contents. These are :—A history of the
use of remedial agents in the case of disease ;—on the origin of
medical experience ; on the theory of the action of medicines ;
on pharmacological nomenclature, &c.; and on the classification of
remedial agents :—after which he considers “ special pharmacody-
namics, treating the different classes in the following order. In
different (dietetical) agents; alcoholic and ethereal; ethereal; oily;
resinous; acrid; alkaloid (narcotic;) bitter; astringent; acid;
alkaline; saline (with alkaline base;) metallic and nonmetallic
(metalloid) elementary. This is followed by a sketch of the most
important agents derived fromihe vegetable kingdom, according to
the natural system, and a general view of remedial agents according
to the physiological system, which he divides and sub-divides as
follows : 1. Cerebro-spinantia, (narcotics of authors.) 2. Stimu-
lantia, (Excitantia, Incitantia, Calefacientia.) 3. Tonica seu
Corroborantia. 4. Emollientia et Demulcentia. 5. Refrigerantia
seu Temperantia. 6. Evacuantia, including Liquefacientia, Dia-
phoretica, Diuretica, Errhina, Sialaloga [Sialagoga ?] Expecto-
rantia, Emetica seu Vomitoria, Cathartica seu Purgativa, Em-
menagoga, Cholagoga seu Bilitica, Ecbolica seu Contractores
Uteri, Acida, Alkalina and Topica.
We need not repeat the observations we made in our last num-
ber on the different classifications adopted, and the defects of the
one on which we then animadverted. The one before us classifies
mercury and iodine under the head of Liquefacientia (verfliissigende
Mittel,} which are defined—“ Medicines that increase the secre-
tions, prevent solidification, and consequently aid the fluid pro-
cesses of the body ; and which, by protracted use, indicate a great
alteration in the functions of assimilation.”
ja. The divisions of the class Cerebro-spinantia appear to us even of
more questionable propriety than those adopted by others. They
are ten in number. 1. Convulsiva (Tetanica) ; 2. Paralysantia,
(e. g. coneia) ; 3. Narcotica seu Stupefacientia, (e. g. aconite) ;
4. Stupefacientia convulsiva (e. g. hydrocyanic acid) ; 5. Con-
vulsiva, which occasion delirium, followed by sleep and stupor.
6. Stupefacientia paralysantia, pupillam, contrahentia (narcotica
hypnotica, e. g. opium) ; 7. Inebriantia seu stupefacientia paraly-
santia (e. g. alcohol); and 8. Delirifacientia, pupillam dilatantia
et pharyngem et laryngem paralysantia (e. g. belladonna) ; 9. Se-
dativa cardiaco-vascularia, (e. g. digitalis) ; 10. Cerebro-spinantia
metallica, in which are included lead, manganese, mercurials
when occasioning Tremor \mercurialis, and the preparations of
arsenic, bismuth, copper, silver and zinc, because “ they affect the
true spinal system as shown by their action in epilepsy and
chorea ” ! ! If the object of classification be to simplify, then does
it fail in its object here,—for the result is obviously confusion
" worse confounded.
The volume is concluded with an alphabetical view of the most
important diseases and morbid conditions for which the different
medicinal agents are recommended—a valuable adjunct to a
treatise on the properties of medicines or Pharmacodynamics.

				

